# Enhanced Anionic Redox Reaction of Na-Layered Li-Containing Mn-Based Cathodes by Cu-Mediated Reductive Coupling Mechanism

**DOI:** 10.3390/nano15120893

**Published:** 2025-06-10

**Authors:** Danyang Li, Can Liu, Shu Zhao, Fujie Li, Hao Li, Chao Wang, Xiu Song Zhao

**Affiliations:** Institute of Materials for Energy and Environment, College of Materials Science and Engineering, Qingdao University, Qingdao 266071, China

**Keywords:** sodium-ion battery, layered Mn-based oxide, anionic redox reaction, reductive coupling mechanism, elemental substitution

## Abstract

Na-layered Li-containing Mn-based cathodes (Na*_x_*Li*_y_*Mn*_1-y_*O_2_, NLMOs) with additional Na^+^ storage ability resulting from the anionic redox reaction (ARR) hold great promise for sodium-ion batteries (NIBs). However, practical applications of NLMOs encounter challenges, such as migration of transition metal Mn, loss of lattice oxygen, and voltage decay during cycling. Here, we show that Cu plays an important role in enhancing the ARR via the reductive coupling mechanism (RCM). Results shows that a Cu^2+^/Fe^3+^ modified NLMO sample delivers a Na^+^ storage capacity as high as 174 mA h g^−1^ at 0.2C, higher than that of a Zn^2+^/Fe^3+^ modified NLMO sample (130 mA h g^−1^) and NLMO (154 mA h g^−1^). Both in situ and ex situ characterization results indicate that the obvious improvement in the electrochemical performance of the Cu^2+^/Fe^3+^ modified NLMO is due to the additional overlaps between the Cu 3d and O 2p orbitals, which is beneficial for the RCM. As a result, the ARR is enhanced so as to increase the Na^+^ storage capacity.

## 1. Introduction

Na-layered Li-containing Mn-based cathodes (Na*_x_*Li*_y_*Mn*_1-y_*O_2_, NLMOs) hold great promise for developing high-energy-density sodium-ion batteries (NIBs) because of the additional Na^+^ storage capacity resulting from the anionic redox reaction (ARR) [1,2]. It is the existence of the Li-O-Na configuration in NLMOs that stimulates lattice oxygen ions (O^2−^) to participate in charge compensation by contributing electrons from O 2*p* orbitals during the ARR [3,4]. Studies have shown that elements Mg [5] and Zn [6] can play the same role as Li does in stimulating the ARR. Furthermore, cationic vacancies presented in Na-layered Mn-based cathode materials have also been found to trigger the ARR [7]. However, the overlap between the energy orbitals of the Mn 3*d* electrons and the O 2*p* electrons in these ARR-active materials is weakened [8,9], causing issues such as over-oxidation of active lattice oxygens [10,11], irreversible loss of molecular O_2_, and migration of Mn [12,13]. These issues lead to structural damages [14], the presence of voltage hysteresis [15], and slow ARR reaction kinetics [16] during battery cycling.

Surface coating [17,18], heteroatom doping [19,20], and elemental substitution [11,21,22,23,24] are common strategies for addressing the above issues. Elemental substitution on the part of Mn by transition metals (TMs) has been shown to be an effective approach to strengthening the overlap between the energy orbitals of the TM 3*d* electrons and the O 2*p* electrons [23]. In addition, such substitutions can also increase the layer distance [22], enhance electron conductivity [23], and minimize the over-oxidation of O^2−^ [11]. Interestingly, some TM-substituted NLMO cathodes [25,26,27] display a reductive coupling mechanism (RCM), which is beneficial for enhancing the reversibility of the ARR. The RCM is a process of electron transfer from lattice oxygen to TM ions during the electrochemical oxidation of lattice oxygens, with benefits for the ARR [28,29,30,31,32]. For example, in a P2-Na_0.8_Cu_0.22_Li_0.08_Mn_0.67_O_2_ cathode reported by Wang et al. [25], the 3*d* orbitals of TM Cu and the non-bonding 2*p* orbitals of the lattice oxygen highly overlap to drive the RCM to occur. In this process, a Cu-(O-O) structure is formed, which greatly accelerates the ARR kinetics. Another example is the improvement in both initial Coulombic efficiency and cycling stability of a P2-Na_2/3_Fe_2/9_Ni_2/9_Mn_5/9_O_2_ cathode because of the RCM induced by Fe [26].

In this work, Cu^2+^/Fe^3+^-modified NLMO with a stoichiometry of Na_0.72_Li_0.16_Cu_0.08_Fe_0.08_Mn_0.68_O_2_ (NLCFMO) and Zn^2+^/Fe^3+^-modified NLMO with a stoichiometry of Na_0.72_Li_0.16_Zn_0.08_Fe_0.08_Mn_0.68_O_2_ (NLZFMO) were synthesized and their Na^+^ storage properties were investigated and compared with NLMO. In comparison with NLMO, NLCFMO exhibits increased Na^+^ storage capacity and improved reversibility of the ARR. In contrast, the electrochemical performance of NLZFMO is worse than that of NLMO. We further analyzed the reasons behind such obvious differences using both ex situ and in situ techniques. Characterization results show that Cu plays an important role in enhancing the ARR via the RCM.

## 2. Materials and Methods

### 2.1. Chemicals

All chemicals, including Na_2_CO_3_ (99%, Aladdin, Shanghai, China), anhydrous LiOH (99%, Aladdin, Shanghai, China), MnO_2_ (99%, Macklin, Shanghai, China), α-Fe_2_O_3_ (99%, Aladdin, Shanghai, China), ZnO (99%, Aladdin, Shanghai, China), and CuO (99%, Macklin, Shanghai, China), were used as received.

### 2.2. Preparation of Samples

The samples studied in this work were synthesized using the solid-state reaction method. The synthesis of the Na-layered Li-containing Mn-based cathode (NLMO) sample with a stoichiometry of Na_0.72_Li_0.24_Mn_0.76_O_2_ is described as the following: 1.0612 g of Na_2_CO_3_, 0.1600 g of anhydrous LiOH, and 1.8024 g of MnO_2_ were mixed in a high-energy ball-milling machine (SPEX 8000M, SPEX SamplePrep, LLC, Metuchen, NJ, USA) for 1 h, followed by thermal treatment in a muffle furnace at 750 °C for 12 h with a heating rate of 5 °C min^−1^ in an air atmosphere. The synthesis of the Cu^2+^/Fe^3+^ pair-modified NLMO sample with a stoichiometry of Na_0.72_Li_0.16_Cu_0.08_Fe_0.08_Mn_0.68_O_2_ (NLCFMO) followed the same precursor as that of NLMO apart from using different quantities of precursors, which were 1.0487 g of Na_2_CO_3_ (in 2% excess in stoichiometry), 0.1047 g of anhydrous LiOH (in 2% excess in stoichiometry), 1.5578 g of MnO_2_, 0.1672 g of α-Fe_2_O_3_, and 0.1443 g of CuO. The synthesis of the Zn^2+^/Fe^3+^ pair-modified sample with a stoichiometry of Na_0.72_Li_0.16_Zn_0.08_Fe_0.08_Mn_0.68_O_2_ (NLZFMO) followed the same procedure as that of NLMO apart from using different quantities of precursors, which were 1.0200 g of Na_2_CO_3_, 0.1024 g of anhydrous LiOH, 0.1707 g of ZnO, 0.1783 g of Fe_2_O_3_, and 1.5501 g of MnO_2_.

### 2.3. Characterization

The crystal structures of the samples were characterized using the X-ray diffraction (XRD) technique on an Ultima IV powder X-ray diffractometer (Rigaku Co., Ltd. Akishima, Japan) with Cu Kα radiation (*λ* = 1.5406 Å) at a scanning speed of 10° min^−1^. The collected XRD patterns were subjected to Rietveld refinement using the Fullprof software (version 5.10 jan-2023). The morphology and elemental composition of the samples were characterized using a field emission scanning electron microscope (FESEM, JSM-7800F, JEOL Ltd. Tokyo, Japan) at an accelerating voltage of 10 kV. The elemental distribution in the samples was characterized using the energy-dispersive spectroscopy accessory (EDS, 51-XMX1236, Oxford Instruments, Oxford, UK) equipped with the FESEM machine. Transmission electron microscope (TEM) images were obtained on a JEM-2100Plus (JEOL) operated at an acceleration voltage of 200 kV. Surface analysis was conducted on an X-ray photoelectron spectrometer (XPS, PHI5000 Ver-saprobe III, ULVAC-PHI, Chigasaki City, Japan) with an aluminum (Al) X-ray source. The elemental analysis of materials was carried out using a inductively coupled plasma optical emission spectrometer (ICP-OES, Thermo Fisher iCAP PRO, Waltham, MA, USA).

### 2.4. Electrochemical Measurement

The electrochemical performance of the samples was tested using CR2032 coin cells (NEWARE TECHNOLOGY LIMITED, Shenzhen, China). An active electrode material, Super P, and polyvinylidene fluoride (PVDF) were mixed in a mass ratio of 8:1:1 to disperse in N-methyl-2-pyrrolidone (NMP). The obtained slurry was coated on aluminum foil, dried at 110 °C for 12 h in a vacuum oven, and cut into discs with a diameter of 12 mm. The mass loading of the active material was measured to be about 1.5 mg cm^−2^. Sodium metal foil was used as the counter electrode and glass fiber (Whatman GF/D, Maidstone, UK) was used as the separator. A 1.0 M measure of NaClO_4_ in a mixture of ethylene carbonate (EC), propylene carbonate (PC), and dimethyl carbonate (DMC) (volume ratio = 1:1:1) with 2 vol% fluoroethylene carbonate (FEC) was used as the electrolyte. All coin cells were assembled in an argon-filled glove box with O_2_ and H_2_O concentrations below 0.1 ppm. Galvanostatic charge–discharge (GCD) analyses were performed on a Neware instrument (CT-4008) in the voltage range between 2.0 and 4.5 V. The cyclic voltammetry (CV) test was performed on a Gamry electrochemical workstation (Interface 1010) in the voltage range between 2.0 and 4.5 V at a scan rate of 0.01 mV s^−1^. The electrochemical impedance spectroscopy (EIS) of electrode materials was performed using a CHI660 electrochemical workstation (CH Instruments Co., Ltd. Shanghai, China), with the frequency scanned from 100 kHz to 0.01 Hz and an amplitude of 5 mV. The Warburg coefficient σ was obtained by linear fitting of *Z*′ versus *ω*^−1/2^. The diffusion coefficient of Na⁺ was calculated using the following formula:

$$D_{{\mathrm{Na}}^{+}}=\frac{R^{2}T^{2}}{2A^{2}n^{4}F^{4}C^{2}\sigma^{2}}$$

where *R* is the gas constant, *T* is the absolute temperature, *A* is the effective electrode area, *n* is the number of transferred electrons in the reaction, *F* is the Faraday constant, and *C* is the concentration of sodium ions. In situ XRD measurements were conducted using a beryllium-equipped customized electrochemical cell (Beijing Scistar Technology Co., Ltd. Beijing, China).

## 3. Results and Discussion

### 3.1. Structure and Morphology

**Figure 1:**
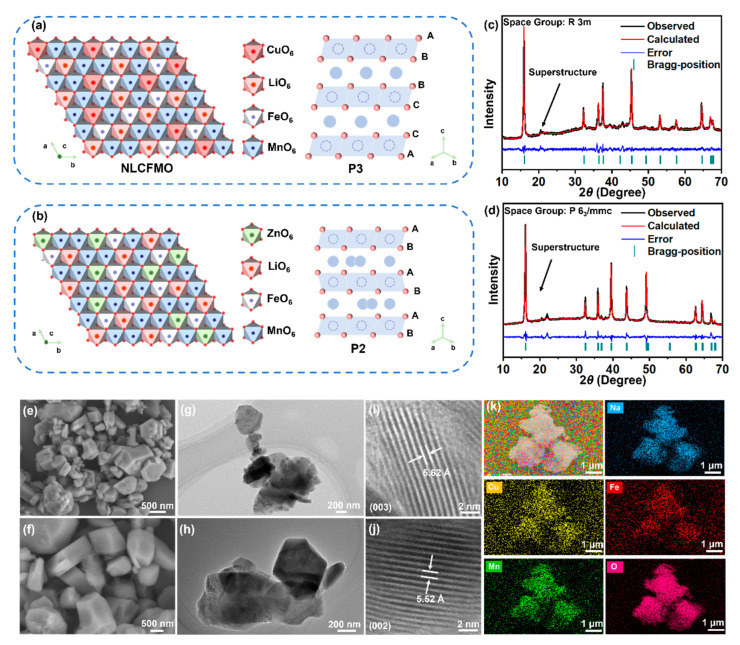
Schematic illustration of the crystal structures of P3-NLCFMO (**a**) and P2-NLZFMO (**b**). Experimental XRD patterns and Rietveld refinement patterns of NLCFMO (**c**) and NLZFMO (**d**). FESEM images of NLCFMO (**e**) and NLZFMO (**f**). TEM images of NLCFMO (**g**) and NLZFMO (**h**). HRTEM images of the (003) crystal plane of NLCFMO (**i**) and (002) crystal plane of NLZFMO (**j**). SEM-EDS mapping images of NLCFMO (**k**).

As is seen from Table S1, the ICP-OES results indicate that the molecular formulae of the two samples are Na_0.70_Li_0.16_Cu_0.077_Fe_0.083_Mn_0.68_O_2_ and Na_0.70_Li_0.15_Zn_0.08_Fe_0.81_Mn_0.68_O_2_, respectively, which are in line with the stoichiometries in the synthesis. Figure 1a,b schematically illustrate the crystal structures of P3-NLCFMO and P2-NLZFMO, respectively. The crystallographic data of NLCFMO and NLZFMO obtained using the XRD Rietveld refinement method are depicted in Figure 1c,d and Tables S2 and S3. It can be observed that NLCFMO exhibits a P3 structure (PDF No. 00-054-0839) with a hexagonal R 3m space group while NLZFMO maintains the P2 structure (PDF No. 00-054-0894) of NLMO with a hexagonal P 6_3_/mmc space group [21,33,34].

The SAED data of Figure S1 further prove the crystal structures of the two samples [22]. The difference in the crystal structure of NLCFMO from that of NLZFMO can be attributed to the stronger Cu-O bond, which needs a higher energy to break, thus inhibiting the transformation from P3 to P2 phases. It is interesting to note that a small peak at around 22° two theta appears on both NLCFMO and NLZFMO, indicating the formation of Li@(Fe/Mn)_6_ and Cu@(Fe/Mn)_6_ superstructures units in NLCFMO and Li@(Fe/Mn)_6_ and Zn@(Fe/Mn)_6_ superstructure units in NLZFMO, respectively [11,12,35]. The existence of such superstructures is beneficial for the reversibility of the ARR [12,36].

Tables S2 and S3 show the crystal structure information of NLCFMO and NLZFMO. Besides the different stacking patterns of O, the main difference between NLCFMO and NLZFMO lies in the occupation sites of Na^+^. Na^+^ only occupies the 3a site in P3-NLCFMO, while Na^+^ occupies the 2b and 2d sites in P2-NLZFMO. The lattice parameters *a* and *b* of NLCFMO (*a* = *b* = 2.8863 Å) are smaller than those of NLZFMO (2.8914 Å). This is due to the larger ionic radius of Zn^2+^ than Cu^2+^ [37,38]. The lattice parameter *c* of NLCFMO (*c* = 5.62 Å) is larger than that of NLZFMO (5.52 Å), which is attributed to the stronger Cu-O bond than Zn-O [39,40]. The effective negative charge around oxygen ions decreases, which weakens the interaction between Na-O bonds, resulting in a longer *c*-axis in NLCFMO [25,41]. This is beneficial for the diffusion of Na^+^ and the enhancement of electrostatic shielding effect between O layers [20,24].

Figure 1e,f show the FESEM images of NLCFMO and NLZFMO; the morphologies of both samples indicate a lamellar structure with particle sizes ranging between 500 and 1000 nm. The particle size distribution curve shown in Figure S2 confirmed the particle size range. Figure 1g,h exhibit the TEM images of NLCFMO and NLZFMO, respectively, further confirming the layered morphology. The HRTEM images of NLCFMO and NLZFMO are shown in Figure 1i and 1j, respectively. The lattice spacing of the (003) plane of NLCFMO is 5.62 Å, which is larger than that of the (002) plane of NLZFMO (5.52 Å), in agreement with the XRD Rietveld refinement results. The increased layer spacing in NLCFMO enhances the kinetics of Na^+^ diffusion. HRTEM images clearly show that Zn^2+^ substitution causes lattice distortion in NLZFMO, which may adversely affect Na^+^ diffusion. Due to the slight difference in ionic radii between Zn^2+^ (0.74 Å) and Cu^2+^ (0.73 Å), it is unlikely to alter the layered crystal structure. Moreover, the *d*_10_ electron configuration of Zn^2+^ cannot effectively overlap with the O 2*p* orbitals, resulting in ionic Zn-O bonds and covalent Cu-O bonds. The strong covalent nature of Cu-O bonds in NLCFMO endows it with strong resistance to lattice distortion, so the lattice fringes of NLCFMO are relatively regular and smooth with fewer lattice distortions. The EDS results in Figure 1k and Figure S3 show uniform distributions of Na, Cu, Zn, Fe, Mn, and O elements, confirming the substitution of Cu^2+^/Fe^3+^ in NLCFMO and Zn^2+^/Fe^3+^ in NLZFMO. The XPS survey spectra of NLCFMO and NLZFMO shown in Figure S4 further demonstrate the presence of Cu and Fe in NLCFMO and the presence of Zn and Fe in NLZFMO.

### 3.2. Electrochemical Performance

**Figure 2:**
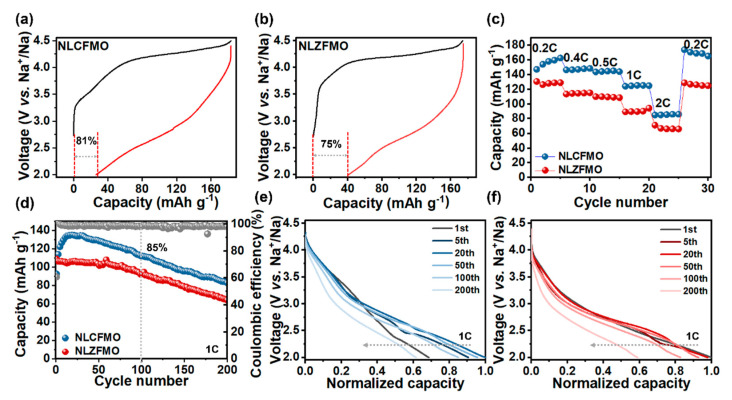
The initial GCD curves of NLCFMO (**a**) and NLZFMO (**b**) measured at 0.2C in the voltage range between 2.0 and 4.5 V. Rate performance of NLCFMO and NLZFMO (**c**). Cycling stability of NLCFMO and NLZFMO measured at 1C (**d**). The normalized discharge curves of NLCFMO (**e**) and NLZFMO (**f**) at 1C.

Figure 2a,b exhibit the initial GCD curves of NLZFMO and NLCFMO at 0.2C (1C = 200 mA h g^−1^) in the voltage range between 2.0 and 4.5 V. Both electrodes exhibit obvious voltage hysteresis in discharge curves, corresponding the characteristic behaviors of ARR triggered by the Li-O-Na and Zn-O-Na configurations [6,35]. NLZFMO shows a lower discharge voltage than that of NLCFMO, which is ascribed to the severer rearrangement of Zn, Fe, and Mn in NLZFMO than that of Cu, Fe, and Mn in NLCFMO [42]. The charge curves of both materials exhibit a sloped region below 4.0 V and a charging plateau above 4.0 V, corresponding to the valence change of the transition metal cations and the oxidation process of lattice oxygen [43]. The oxidation of Cu^2+^ to Cu^3+^ extends the sloping charge curve of NLCFMO, which is beneficial for the reduction of voltage hysteresis [44]. Furthermore, NLCFMO shows a higher Coulombic efficiency (81%) than NLZFMO (75%) [26,45]. This may be attributed to the relatively strong covalent nature of the Cu-O bond, which suppresses the irreversible release of lattice oxygen in the first cycle, thus greatly reducing the occurrence of oxygen vacancies.

Figure S5a shows the galvanostatic charge–discharge (GCD) curves of NLMO at 0.2C. The highest specific capacity of 154 mA h g^−1^ is achieved in the first cycle, and the specific capacity decays to 145 mA h g^−1^ in the second cycle. Figure 2c compares the rate performance of NLCFMO and NLZFMO. The NLCFMO electrode maintains reversible discharge capacities of 174, 148,145, 125, and 86 mA h g^−1^ at 0.2C, 0.4C, 0.5C, 1C, and 2C, respectively, higher than that of the NLZFMO electrode. Upon reverting the rate back to 0.2C, the discharge capacity of the NLCFMO electrode is increased to 174 mAh g^−1^, indicating this electrode needs an activation process to reach the highest Na^+^ storage capacity. The high specific capacity of NLCFMO is related to the RCM triggered by Cu, which enhances the ARR in NLCFMO. The GCD curves of NLZFMO and NLCFMO at different rates depicted in Figure S6 further confirms the improved electrochemical performance of NLCFMO due to the enhanced RCM.

Figure S7 displays the Nyquist plots of NLCFMO and NLZFMO. After fitting with circuit data, the charge transfer resistances of NLCFMO and NLZFMO were calculated to be 36.19 and 57.89 Ω, respectively, indicating that the presence of Cu effectively reduces the material’s resistance to electron transport. It is likely that the energy levels of the Cu d-orbitals reduce the bandgap of NLMO, making it easier for electrons to be excited from the valence band to the conduction band. Calculations show that the Na^+^ diffusivity (*D*_Na+_) of NLCFMO is higher than that of NLZFMO, which is related to the larger interlayer spacing of the former than that of the latter. On the other hand, the severer lattice distortion of NLZFMO could hinder the diffusion pathway of Na⁺, thereby lowering its *D*_Na+_ value.

Figure 2d shows the cycling performance of NLCFMO and NLZFMO measured at 1C. It can be seen that NLCFMO retains 85% capacity after 100 cycles, which is higher than that of NLZFMO (83%). At 1C, the first-cycle efficiency of NLCFMO is 62%. Due to the capacity increase, the Coulombic efficiency in the subsequent few cycles is 102%, and it remains at about 99% in the subsequent cycles. It is noted that the specific capacity of NLCFMO gradually increased in the first 30 cycles, from the initial 92 mA h g^−1^ to 135 mA h g^−1^ at the 30th cycle. This indicates that the electrode material needs to be activated in order to maximize its charge storage capacity. After reaching the highest specific capacity of 110 mA h g⁻¹ in the first cycle, NLZFMO shows a continuous decay trend during the subsequent cycles. Figure S5b shows the cycling performance of NLMO measured at 1C. The initial specific capacity is about 89 mA·h·g^−1^ in the first cycle, reaching a maximum of 110 mA·h·g^−1^ after 50 cycles, and then declining continuously. After 100 cycles, the capacity retention is 87%. These materials exhibit relatively short cycle life, which may be attributed to multiple factors such as the accumulation of irreversible phase transitions during cycling [14,20] and irreversible reactions between high-valent oxygen and electrolyte during the ARR process [46]. Figure 2e,f display the normalized capacity discharge curves of NLCFMO and NLZFMO, further illustrating that NLCFMO has better cycling stability compared to NLZFMO. The discharge voltages of both materials decrease during the cycling process, and the degree of decrease for NLZFMO is more drastic, which is related to the continuous destruction and reorganization of the superstructure of the material. The RCM of Cu enhances the interaction of the Cu-O bonds and suppresses the irreversible release of oxygen.

### 3.3. Structural Evolution During Cycling

**Figure 3:**
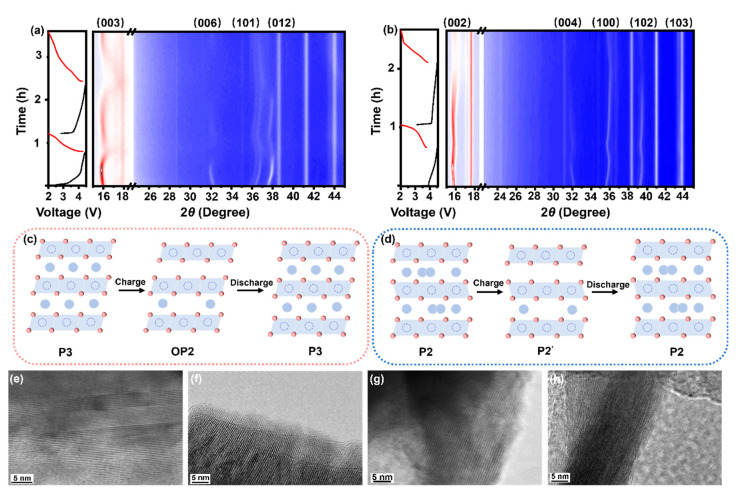
In situ XRD contour plot of (**a**) NLCFMO and (**b**) NLZFMO. The corresponding phase transition during cycling of (**c**) NLCFMO and (**d**) NLZFMO. Ex situ HRTEM images of NLCFMO in the pristine state (**e**) and after 50 cycles (**f**). Ex situ HRTEM images of NLZFMO in the pristine state (**g**) and after 50 cycles (**h**).

In situ XRD measurements in the first two cycles at 0.5C for NLCFMO and NLZFMO electrodes were performed to study their structural evolution during cycling, and the results are shown in Figure 3a,b. As is seen from Figure 3a, at the beginning of charging, the main (003) peak and the (006) peak of NLCFMO first shifted to lower angles, indicating an increase in the inter-layer spacing along the *c*-axis [47]. This is because when Na^+^ is extracted from the interlayer, the electrostatic shielding effect from the two TM layers is weakened [48]. When charged to 4.1 V, the (003) and (006) peaks continued to shift to higher angles two theta, and new peaks attributed to the OP2 phase appears (Figure 3c) [49]. As the phase transition occurs, the (006) peak is broadened and eventually disappears. The (101) and (012) peaks always shifted to higher angles during the charging process, demonstrating that the TM layer contracts along the *a* and *b* axes, which is due to the oxidation of Cu^2+^/Cu^3+^ and Fe^3+^/Fe^4+^ [50]. During the sodiation process, the (003) and (006) peaks appears again, demonstrating that the P3 phase is recovered from the OP2 phase reversibly (Figure 3c) [49]. At the second cycle, the diffraction peaks representing the OP2 phase are more obvious above 4.0 V, confirming that the phase transition process of P3→OP2→P3 is highly reversible. This phase transition in NLCFMO can alleviate the accumulation of mechanical stress in the electrode.

Figure 3b shows the in situ XRD curve of NLZFMO. At the beginning of the charging process, the (002) and (004) peaks shift to lower angles, demonstrating an increase in the interlayer spacing along the *c*-axis [20]. This is related to the weakening of the electrostatic shielding effect, which is similar to NLCFMO. The (100) and (102) peaks shift to higher angles, corresponding to the decrease in interlayer spacing along the *a* and *b* axes, which is caused by the changes in cation valence [14]. When charged to 4.2 V, the (002) peak shifts to a higher angle, which means the contraction of the *c*-axis. This is related to the enhanced shielding effect between the upper and lower O layers during the ARR process [48]. The broadening and disappearance of the (004) peak indicates that the P2 phase transforms into the P2’ phase (Figure 3d) [51]. During the second cycle, the intensity of the (002) peak became obviously weak when charged to 4.5 V, revealing the unstable structure of NLZFMO.

To further verify the structural stability of NLCFMO and NLZFMO against cycling, HRTEM measurements were performed on both electrodes after 50 cycles. The results are shown in Figure 3h. As can be seen from Figure 3e, the (003) crystal plane of the pristine state NLCFMO exhibits clear and intact lattice fringes. After 50 cycles, its lattice fringes are well preserved with only partial crystal plane slip and lattice distortion (Figure 3f). This phenomenon can be attributed to the reversible phase transition in NLCFMO, which is favorable for maintaining the structural stability against cycling. Additionally, the structural stability of NLCFMO may also benefit from the strong covalent Cu-O bonds. In contrast, the lattice fringes of the (002) crystal plane in NLZFMO became more fragmented and blurred after 50 cycles (Figure 3h), indicating substantial crystal plane slip and lattice distortion. Compared to the strong covalent Cu-O bond, the covalent Zn-O bond tends to be of ionic nature. This difference leads the NLZFMO structure to be less stable than NLCFMO against cycling.

### 3.4. Charge Compensation Mechanism

**Figure 4:**
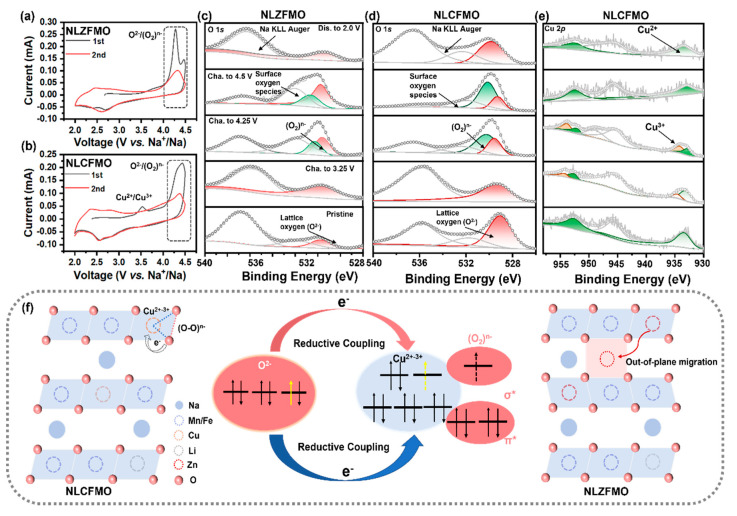
CV curves of (**a**) NLZFMO and (**b**) NLCFMO. Ex situ O 1*s* XPS spectra of (**c**) NLZFMO and (**d**) NLCFMO. (**e**) Ex situ Cu 2*p* XPS spectra of NLCFMO. (**f**) Schematic illustrations of the reductive coupling mechanism of NLCFMO and ion migration in NLZFMO.

Figure 4a,b show the CV curves of the NLCFMO and NLZFMO electrodes for the early two cycles measured at 0.1 mV s^−1^ in the voltage range between 2.0 and 4.5 V. Both electrodes exhibit sharp oxidation peaks above 4.0 V, corresponding to the ARR process. NLZFMO shows divisive peaks in the range between 4.0 and 4.5 V, which is related to the high and low states of O 2*p* non-bonding orbital energy levels [11,37]. It is worth noting that the peak current of NLZFMO is higher than that of NLCFMO. Because the Zn-O-Na configuration can also excite the ARR, the peak current attributed to O^2−^ oxidation is higher. However, electrochemical performance results show that NLZFMO has lower specific capacity and ICE, indicating poorer reversibility, which is probably caused by severer oxygen evolution reaction (OER) [3,44]. In contrast, in NLCFMO, except for Li, there are no additional elements that can excite the ARR. Nevertheless, an important reason for its highest specific capacity is due to the RCM induced by Cu, which enhances the reversibility of the ARR, leading to unfavorable OER. There is no obvious redox pair of Mn^3+^/Mn^4+^ for both NLCFMO and NLZFMO electrodes, which can effectively suppress the Jahn–Teller effect caused by Mn^3+^ [52,53]. The relatively broad redox pair between 2.5-3.0 V comes from Fe^3+^/Fe^4+^ redox reactions. In the CV curve of NLCFMO, a sharp oxidation peak belonging to Cu^2+^/Cu^3+^ appears at around 3.5 V [44,54]. There is no sharp reduction peak belonging to Cu^3+^/Cu^2+^ during the reduction process, which is ascribed to the strong RCM of Cu. During the ARR process, there is a strong orbital overlap between Cu and O. The oxidized high-valence oxygen ions transfer electrons to the central metal Cu, leading to a large amount of Cu^3+^ being reduced before discharging [25].

Figure 4c,d show the O 1*s* XPS spectra during different charging and discharging processes of the NLZFMO and NLCFMO electrodes, respectively. For the pristine electrodes, three peaks at 529.4, 532.0, and 537.3 eV can be seen from both electrodes, corresponding to lattice oxygen O^2−^ (red), surface O species, and Na KLL Auger electrons, respectively [6,55]. Upon charging over 4.0 V, a new peak at 530.5 eV (green) appears for both NLZFMO and NLCFMO electrodes. This peak is attributed to (O_2_)^n−^ [25,56]. Upon further charging, the intensity of the (O_2_)^n−^ peak is enhanced and the position is shifted slightly towards higher binding energy, indicating the oxidation reaction of O^2−^/(O_2_)^n−^ [37,57]. NLCFMO exhibits a higher (O_2_)^n−^ peak than that of NLZFMO, demonstrating more ARR due to the strong RCM of Cu.

Figures S8 and S9 show the Mn 2*p* XPS spectra of NLCFMO and NLZFMO, respectively. During cycling, there are only two peaks at 653.9 and 642.2 eV, corresponding to Mn2*p*_3/2_ and Mn2*p*_1/2_ of Mn^4+^, respectively [18,58]. This further confirms that Mn^4+^ does not participate in charge compensation during the charge–discharge processes, in agreement with the CV results. Figures S10 and S11 show the ex situ XPS spectra of Fe 2*p* of NLCFMO and NLZFMO, respectively. Due to the interference of the C signal, only the Fe2*p*_1/2_ spectrum can be detected. The purple peak at 711.3 eV represents Fe^3+^. When charged to 3.25 V, a new peak (in yellow) at 713.3 eV due to Fe^4+^ appears [26]. As Na^+^ is extracted, the proportion of the yellow peak gradually increases. However, during the charging process, the proportion of Fe^3+^ always remains relatively high, and it returns to its initial position after the discharge is completed, indicating that all iron ions are reduced back to Fe^3+^. Figure 4e shows the ex situ XPS spectrum of Cu of NLCFMO. In the pristine state, there are two peaks at 933.1 and 952.7 eV, corresponding to Cu2*p*_3/2_ and Cu2*p*_1/2_ peaks of Cu^2+^ (green) [25,44]. When charged to 3.25 V, the Cu2*p*_3/2_ (934.5 eV) and Cu2*p*_1/2_ (953.7 eV) peaks of Cu^3+^ (red) appeared and were continuously enhanced during the charging process to 4.25 V. However, when charging to 4.5 V, the Cu^3+^ peaks almost disappeared. This reverse behavior provides experimental evidence for the metal-induced reduction-coupling mechanism of Cu.

Figure 4f schematically illustrates the ARR mechanism of NLCFMO and NLZFMO. CV and ex situ XPS results confirm that Mn^4+^ hardly participates in the charge compensation of both samples, attributed to the relatively low energy level of the Mn 3*d* orbital. In NLZFMO, the overlap between high energy level O 2*p* orbitals and TM *d* orbitals is minimal, potentially leading to more irreversible reactions during charging. Additionally, Zn^2+^, similar to Li^+^, undergoes thermodynamically favorable in-plane and out-of-plane migration, but with poorer reversibility. Such irreversible ion migration typically promotes excessive oxidation of O^2-^ in ARR, causing the formation of superoxide groups and oxygen evolution reactions in NLZFMO’s ARR process. These side reactions and by-products disrupt the crystal structure stability of NLZFMO, serving as a key factor for its deteriorated electrochemical performance.

During the charging of NLCFMO, analogous to NLZFMO, the Fe^3+^/Fe^4+^ redox couple with a lower working potential emerges first. Upon further charging, electrons are lost from the higher-energy Cu 3*d* orbitals, oxidizing Cu^2+^ to Cu^3+^, after which ARR participates in charge compensation. Once the active lattice oxygen ions (O^2-^) are oxidized, the high-valent lattice oxygen is thermodynamically unstable. Here, the strong overlap between Cu 3*d* and O 2*p* orbitals triggers RCM. During charging, to form thermodynamically more stable peroxo-like bonds, lattice O ions transfer extra electrons to the energy level of Cu 3*d* orbitals, reducing the oxidized Cu^3+^ back to Cu^2+^. This process induces distortion of the CuO_6_ octahedron, where high-valent oxygens form thermodynamically stable (O-O) weak bonds, creating peroxide-like species (O_2_)^n-^ and thus a Cu-(O-O) structure. The RCM effectively suppresses excessive oxidation of O and stabilizes ARR. In Figure 4e, the reduced Cu^2+^ at the 4.5 V state is clearly observed, while Fe remains predominantly in the tetravalent state at 4.5 V. This confirms that Fe does not undergo RCM, indicating that the main role of Fe is similar to that of Mn^4+^, serving as the basic structural framework of the material. For NLMO and NLZFMO, although the Zn-O-Na and Li-O-Na configurations theoretically enhance ARR to improve Na^+^ storage capacity, the extremely stable electronic configuration (*d*_10_) of Zn^2+^ in NLZFMO prevents it from acquiring electrons from O 2*p* orbitals. Consequently, RCM does not occur during ARR, leading to a relatively lower Na^+^ specific storage capacity in NLZFMO.

## 4. Conclusions

Utilizing the anionic redox reaction in Na-layered Li-containing Mn-based cathode materials is a feasible approach to enhancing the energy density of Na^+^ batteries. This work shows that Cu is a unique metal for triggering the anionic redox reaction via a reductive coupling mechanism. Characterization results show that electron transfer from lattice oxygen to Cu ions is enhanced due to additional overlay of Cu 3*d* and O 2*p* orbitals. In addition, the presence of Cu in Na-layered Li-containing Mn-based cathode materials expands the (003) interlayer spacing to facilitate Na^+^ diffusion and accommodate structural distortion during the anionic redux reaction. A Cu-(O-O) structure in a Cu^2+^/Fe^3+^ modified Li-containing Mn-based cathode is formed through the reductive coupling mechanism. This structure can effectively enhance the anionic redox reaction and inhibit the over-oxidation of lattice oxygen. As a result, this Cu^2+^/Fe^3+^ modified Li-containing Mn-based cathode delivers a Na^+^ storage capacity as high as 174 mA h g^−1^ at 0.2C, much higher than that of the parent counterpart electrode and a Zn^2+^/Fe^3+^ modified Li-containing Mn-based cathode.

## Data Availability

Data are contained within the article and Supplementary Materials.

## Supplementary Materials

The following supporting information can be downloaded at: https://www.mdpi.com/article/10.3390/nano15120893/s1, Figure S1: SAED patterns of (a) NLCFMO and (b) NLZFMO viewed from the [100] direction; Figure S2: Particle size distribution of NLCFMO measured using the dynamic light scattering technique; Figure S3: SEM-EDS mapping images of NLZFMO; Figure S4: XPS spectra of (a) NLZFMO and (b) NLCFMO; Figure S5: GCD curves measured at 0.2C (a) and cycling stability (b) of NLMO measured at 1C; Figure S6: GCD curves of (a) NLZFMO and (b) NLCFMO measured at different C-rates; Figure S7: Nyquist plots and fitting curves of NLCFMO and NLZFMO. The insets show the corresponding equivalent circuits, charge-transfer resistances, and Na^+^ diffusivity; Figure S8: ex situ Mn 2*p* XPS spectra of NLCFMO at different charging/discharging states; Figure S9: ex situ Mn 2*p* XPS spectra of NLZFMO at different charging/discharging states; Figure S10: Ex situ Fe 2*p* XPS spectra of NLCFMO at different charging/discharging states; Figure S11: Ex situ Fe 2*p* XPS spectra of NLCFMO at different charging/discharging states; Table S1: Molar composition of Inductively coupled plasma-optical emission spectrometry (ICP-OES) results for NLMO and NLNFMO; Table S2: Rietveld refinement results of the XRD pattern for NLCFMO; Table S3: Rietveld refinement results of the XRD pattern for NLZFMO.
