# Supplementary material for: Enhanced Anionic Redox Reaction of Na-Layered Li-Containing Mn-Based Cathodes by Cu-Mediated Reductive Coupling Mechanism
Source: Nanomaterials (Basel). 2025 Jun 10;15(12):893. doi: 10.3390/nano15120893 (PMC12196220; doi:10.3390/nano15120893)
Supplement: Supplementary file 1 [file nanomaterials-15-00893-s001.zip › nanomaterials-3645841-supplementary.pdf]

Supplementary Materials

Enhanced Anionic Redox Reaction of Na-Layered Li-Containing Mn-Based Cathodes by Cu-Mediated Reductive Coupling Mechanism

Danyang Li, Can Liu, Shu Zhao, Fujie Li, Hao Li and Chao Wang * and Xiu Song Zhao *

Institute of Materials for Energy and Environment, College of Materials Science and Engineering, Qingdao University, Qingdao 266071, China; lidanyang@qdu.edu.cn (D.L.); liucan@qdu.edu.cn (C.L.); zhaoshu@qdu.edu.cn (S.Z.); lifujie@qdu.edu.cn (F.L.); lihao15@qdu.edu.cn (H.L.)

* Correspondence: wangc@qdu.edu.cn (C.W.); chezxs@qdu.edu.cn (X.Z.)

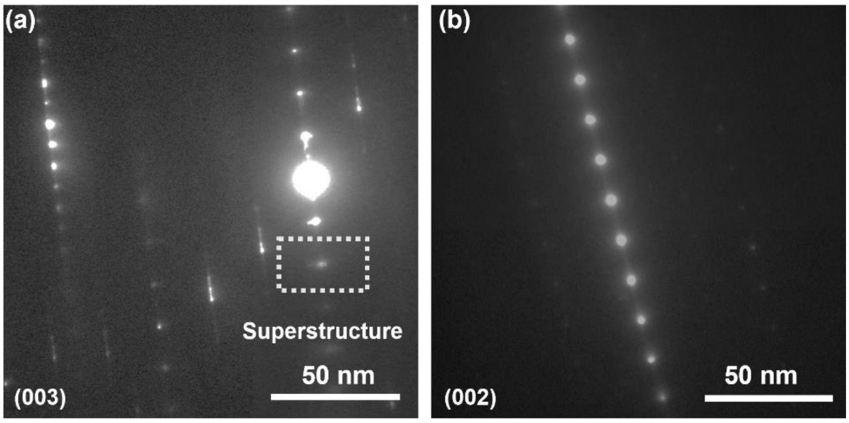

Figure S1. SAED patterns of (a) NLCFMO and (b) NLZFMO viewed from the [100] direction.

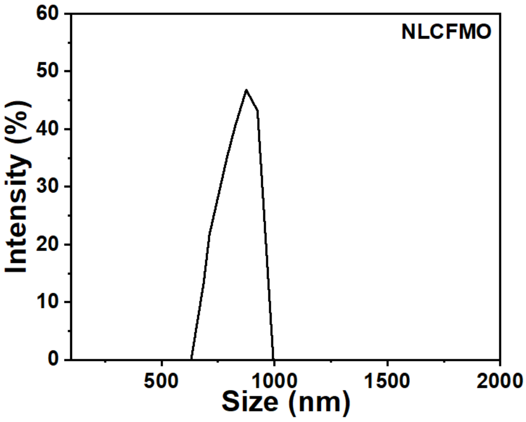

Figure S2. Particle size distribution of NLCFMO measured using the dynamic light scattering technique.

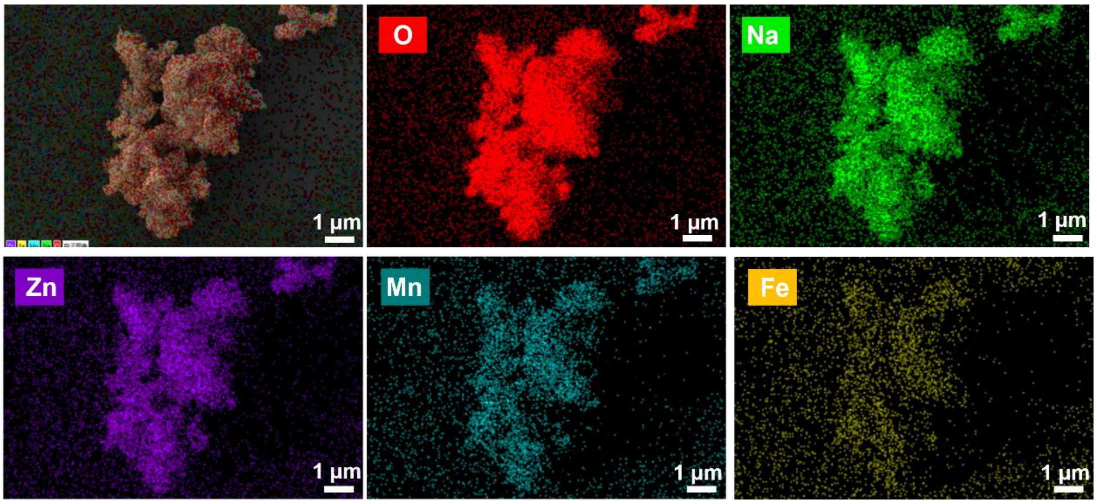

Figure S3. SEM-EDS mapping images of NLZFMO.

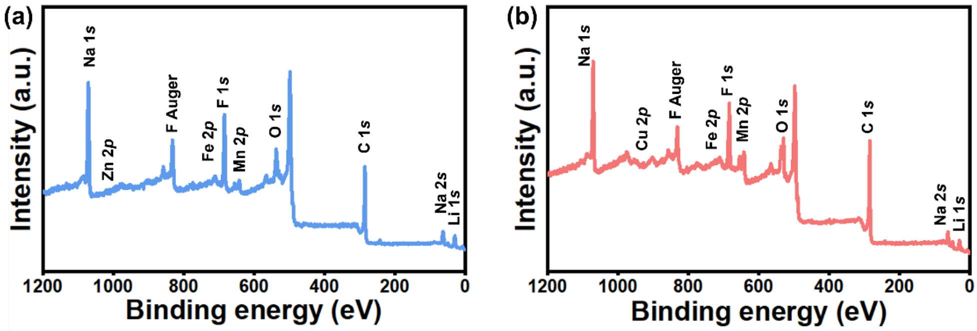

Figure S4. XPS spectra of (a) NLZFMO and (b) NLCFMO.

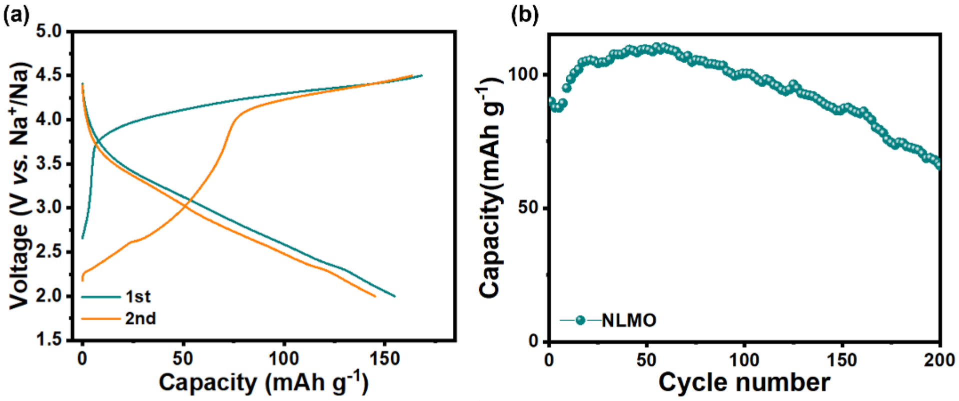

Figure S5. GCD curves measured at 0.2C (a) and cycling stability (b) of NLMO measured at 1C.

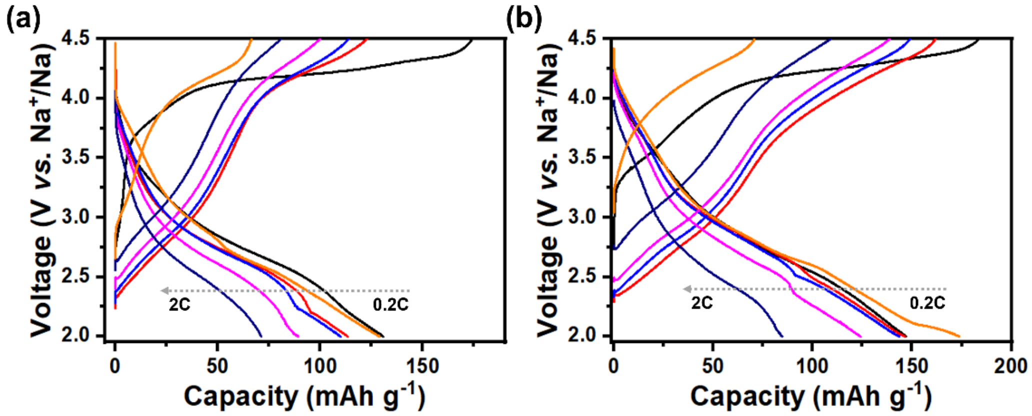

Figure S6. GCD curves of (a) NLZFMO and (b) NLCFMO measured at different C-rates.

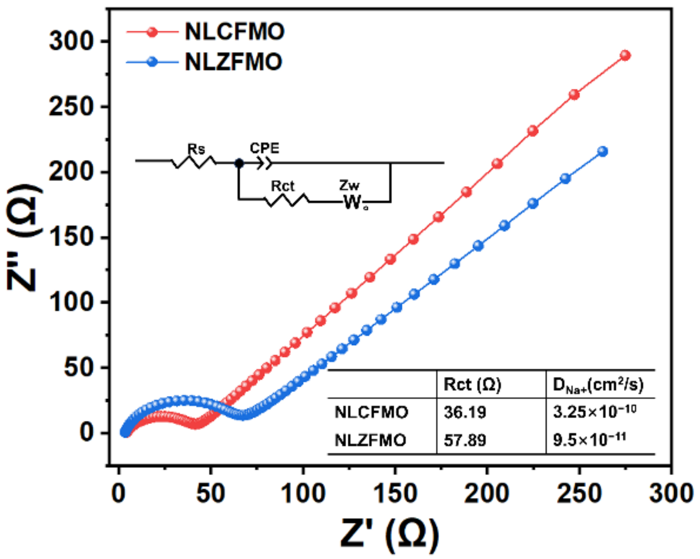

Figure S7. Nyquist plots and fitting curves of NLCFMO and NLZFMO. The insets show the corresponding equivalent circuits, charge-transfer resistances, and Na^+ diffusivity.

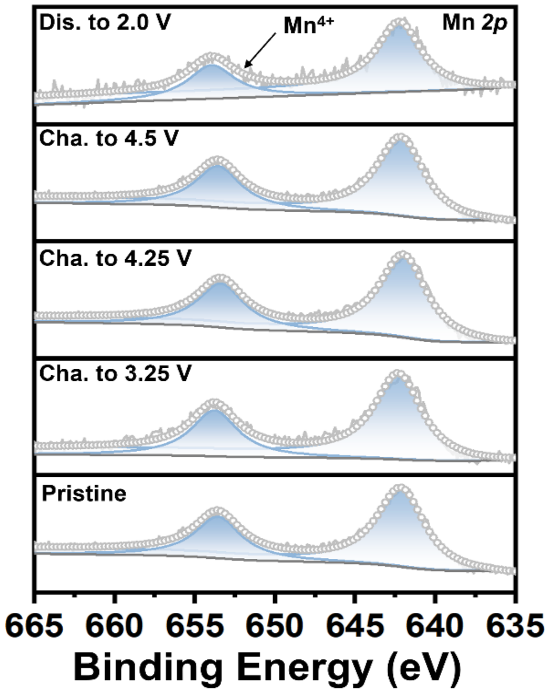

Figure S8. ex situ Mn 2*p* XPS spectra of NLCFMO at different charging/discharging states.

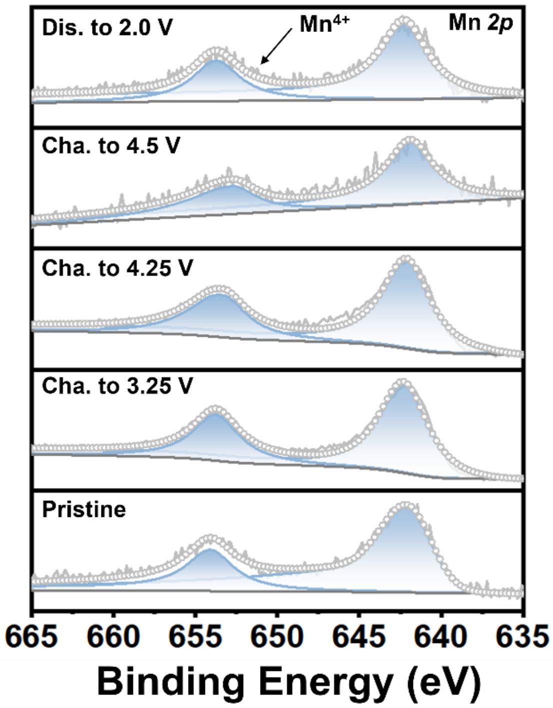

Figure S9. ex situ Mn 2*p* XPS spectra of NLZFMO at different charging/discharging states.

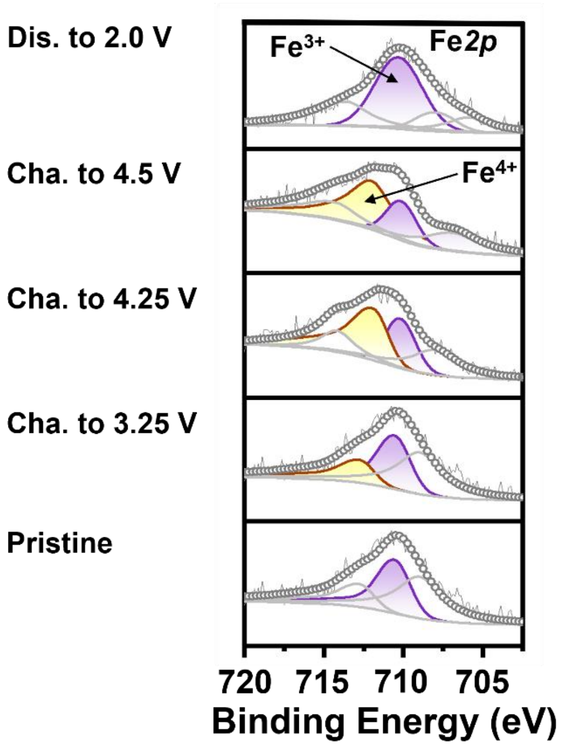

Figure S10. Ex situ Fe 2*p* XPS spectra of NLCFMO at different charging/discharging states.

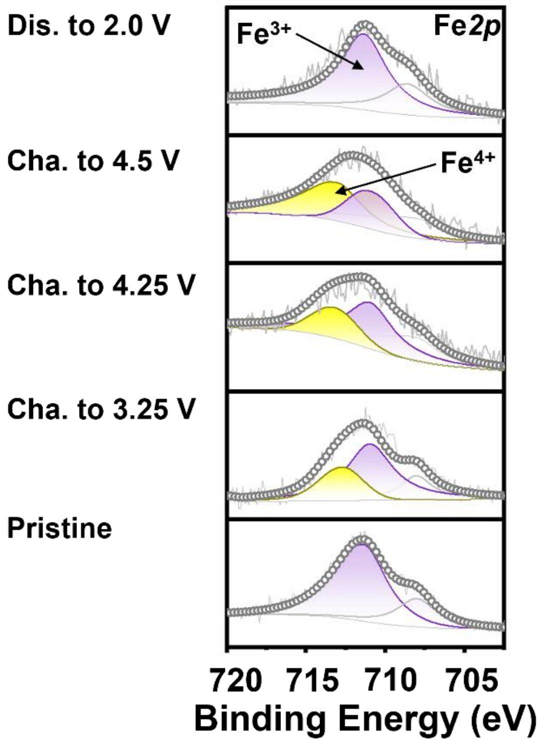

Figure S11. Ex situ Fe 2*p* XPS spectra of NLCFMO at different charging/discharging states.

Supplementary Tables

Table S1. Molar composition of Inductively coupled plasma-optical emission spectrometry (ICP-OES) results for NLMO and NLNFMO.

Samples	Measured atomic radio by ICP-OES					
	Na	Li	Cu	Zn	Fe	Mn
$Na_{0.70}Li_{0.16}Cu_{0.077}Fe_{0.083}Mn_{0.68}O_2$	0.702	0.156	0.077	\	0.0826	0.681
$Na_{0.70}Li_{0.15}Zn_{0.08}Fe_{0.81}Mn_{0.68}O_2$	0.704	0.154	\	0.084	0.809	0.683

Table S2. Rietveld refinement results of the XRD pattern for NLCFMO.

Atom	Site	x	y	z	g (occupancy)
Na	3a	0.00000	0.00000	0.42000	0.720
Li	3a	0.00000	0.00000	0.26000	0.160
Mn	3a	0.00000	0.00000	0.26000	0.680
Cu	3a	0.00000	0.00000	0.26000	0.080
Fe	3a	0.00000	0.00000	0.26000	0.080
O1	3a	0.00000	0.00000	0.63000	1.00
O2	3a	0.00000	0.00000	0.85000	1.00
NLCFMO space group: R 3m					
$a = b = 2.8863$ Å, $c = 16.8464$ Å, $V = 120.1033$ Å$^{\wedge 3}$					

Table S3. Rietveld refinement results of the XRD pattern for NLZFMO.

Atom	Site	x	y	z	g (occupancy)
Na1	2d	0.66667	0.33333	0.25000	0.536
Na2	2b	0.00000	0.00000	0.25000	0.295
Li	2a	0.00000	0.00000	0.00000	0.155
Mn	2a	0.00000	0.00000	0.00000	0.676
Cu	2a	0.00000	0.00000	0.00000	0.083
Fe	2a	0.00000	0.00000	0.00000	0.083
O	4f	0.33333	0.66667	1.00000	1.000
NLZFMO space group: P 6_3/mmc					
$a = b = 2.8914$ Å, $c = 11.0481$ Å, $V = 79.9898$ Å$^{\wedge 3}$					
